# Ca_v_1.4 congenital stationary night blindness is associated with an increased rate of proteasomal degradation

**DOI:** 10.3389/fcell.2023.1161548

**Published:** 2023-05-03

**Authors:** Tal T. Sadeh, Richard A. Baines, Graeme C. Black, Forbes Manson

**Affiliations:** ^1^ Division of Evolution, Infection and Genomics, Faculty of Biology, Medicine and Health, University of Manchester, Manchester, United Kingdom; ^2^ Division of Neuroscience, Faculty of Biology, Medicine and Health, University of Manchester, Manchester, United Kingdom; ^3^ Manchester Centre for Genomic Medicine, Manchester Academic Health Sciences Centre, Manchester University NHS Foundation Trust, St Mary’s Hospital, Manchester, United Kingdom

**Keywords:** Ca_v_1.4, CACNA1, voltage-gated calcium channel, variant pathogenicity, proteasome inhibitor, bortezomib

## Abstract

Pathogenic, generally loss-of-function, variants in *CACNA1F*, encoding the Ca_v_1.4α_1_ calcium channel, underlie congenital stationary night blindness type 2 (CSNB2), a rare inherited retinal disorder associated with visual disability. To establish the underlying pathomechanism, we investigated 10 clinically derived *CACNA1F* missense variants located across pore-forming domains, connecting loops, and the carboxy-tail domain of the Ca_v_1.4α subunit. Homology modeling showed that all variants cause steric clashes; informatics analysis correctly predicted pathogenicity for 7/10 variants. *In vitro* analyses demonstrated that all variants cause a decrease in current, global expression, and protein stability and act through a loss-of-function mechanism and suggested that the mutant Ca_v_1.4α proteins were degraded by the proteasome. We showed that the reduced current for these variants could be significantly increased through treatment with clinical proteasome inhibitors. In addition to facilitating clinical interpretation, these studies suggest that proteasomal inhibition represents an avenue of potential therapeutic intervention for CSNB2.

## Introduction

Voltage-gated calcium channels (VGCCs) are integral membrane proteins that regulate calcium influx from the extracellular space. They activate through membrane depolarization that gates the channel open, permitting calcium influx along an electrochemical gradient (Catterall et al., 2005). There are 10 VGCCs, grouped by the α subunit’s voltage sensitivity, with L-type channels (Ca_v_1.1–1.4) being high-voltage channels (Striessnig et al., 2004). Pathogenic variation of all four Ca_v_1 channels is associated with human diseases across a wide range of pathologies, including cardiovascular (Antzelevitch et al., 2007), neurological (Tumienė et al., 2018), psychiatric (Torrico et al., 2019), and retinal diseases (Strom et al., 1998), reflecting the expression patterns of the encoding genes to a considerable degree.

Congenital stationary night blindness type 2 (CSNB2), also known as incomplete CSNB, is a static X-linked inherited retinal disorder that results in nystagmus and reduced visual acuity in affected individuals. Combined molecular and phenotypic testing is essential for the diagnosis of CSNB2 that is caused by pathogenic variants in the *CACNA1F* gene, encoding the Ca_v_1.4α_1_ calcium channel. Ca_v_1.4α_1_ sustains continuous calcium-dependent glutamate release between retinal photoreceptors and bipolar cells. More than 200 CSNB2 *CACNA1F* variants have been reported, including both missense (*n* = 96) and small insertion–deletion (indel; *n* = 67) variants (Boycott et al., 2000; Striessnig et al., 2004; Zeitz et al., 2015; Men et al., 2017).

The mechanisms through which missense mutations act to disrupt each Ca_v_1 channel vary and include both gain of function (GoF) and loss of function (LoF) (Sadeh et al., 2021). Across the spectrum of these disorders, however, it remains unclear whether the phenotypes arise from decreased protein expression, protein instability, or changes to channel kinetics (Hoda et al., 2005; Burtscher et al., 2014). While many studies have sought to establish whether pathogenic variants can be grouped by structural features, residue properties, or topology (Brunklaus et al., 2014; Heyne et al., 2020; Indelicato and Boesch, 2021; Sadeh et al., 2021), it remains the case that a majority of missense mutations, which are identified in a clinical context, lack sufficient evidence to confirm pathogenicity. This frequently results in inappropriate designation (Hosseini et al., 2018) and/or in variants being reported as variants of uncertain significance (VUS), precluding the provision of a molecular diagnosis to patients and families (Hoda et al., 2005). These limitations hinder the development of therapies and highlight that a refined pipeline is required to identify and classify clinically relevant pathogenic variants.

In this study, we selected 10 novel *CACNA1F* VUS from patients who lack a molecular diagnosis and have a putative diagnosis of CSNB2. We confirmed a number of informatics predictions and advanced the understanding of the underlying pathogenic mechanism. We established that all the pathogenic missense variants under study act by LoF, as evidenced by a combination of reduced channel current, protein stability, and channel expression. We further showed that the reduced current for these variants can be significantly increased through treatment with clinical proteasome inhibitors, thus identifying a target pathway for possible therapeutic intervention.

## Materials and methods

### Experimental design

In this study, we predicted the effects of *CACNA1F* variants *in silico* and empirically tested the predictions to improve the interpretation of clinical variants that alter current amplitudes. We investigated 10 *CACNA1F* missense variants using the gold standard whole-cell patch-clamp analysis to quantify current amplitude as a measure of channel function and Western blotting for protein expression. We selected previously used inhibitors to identify the pathways that degrade mutant proteins and tested their potency in rescuing protein expression and current amplitude. A minimum of three biological replicates were used in this study.

### Samples

We selected 10 novel *CACNA1F* VUS from patients that lack a molecular diagnosis and have a putative CSNB2 diagnosis. Such patients were previously tested at the Manchester Genomic Diagnostic Laboratory (MGDL).

### Informatics analysis

#### Physicochemical analysis

The physicochemical properties of each mutation were manually analyzed using NCBI’s Amino Acid Explorer tools (https://www.ncbi.nlm.nih.gov/Class/Structure/aa/aa_explorer.cgi). The “Structure and Chemistry” and “Common Substitutions” tools were used to compare specific physicochemical constraints of the amino acid pair, such as a change in amino acid size, charge, and hydrophobicity. The latter tool relies on the BLOSUM62 matrix to sort the frequency of the substitution.

#### Population database search (gnomAD)

The minor allele frequencies (MAFs) of *CACNA1F* VUS analyzed in this study were searched in the general population on gnomAD (Karczewski et al., 2019). The MAF is the number of times a variant allele occurs in a population for any data set.

#### *CACNA1F* homology model

To analyze the distribution of mutations across channel domains, a computational model was generated. As the crystallography structure of human *CACNA1F* has not been resolved, a homology model was generated. The CACNA1F protein sequence (accession number: O60840.2) was uploaded to the SWISS-MODEL protein structure homology modeling server (Waterhouse et al., 2018). This identified the homologous rabbit *CACNA1S* (Ca_v_1.1) structure generated by cryo-electron microscopy (PDB ID: 5GJV) that has 85% protein sequence homology to human (Wu et al., 2016). PyMOL was used for model visualization (The PyMOL Molecular Graphics System, Version 2.0 Schrödinger, LLC). This is consistent with the approach and structure used by CACNA1F-vp.

CACNA1F-vp pathogenicity prediction analysis and comparison with other tools were performed as described in Sallah et al. (2020).

### Cloning

Mutations were generated by directional sub-cloning into the wild-type *CACNA1F* plasmid and verified by DNA sequencing (Source Bioscience, https://www.sourcebioscience.com). Fragments containing the single-base substitutions were designed and ordered from GeneArt (Thermo Fisher Scientific, United Kingdom). Mutant fragments were cloned into a pUC18 vector with unique restriction enzymes to allow their excision with complementary ends to the wild-type recipient vector. Restriction sites were digested in the mutant and the wild-type vectors and separated by electrophoresis. The corresponding fragments were excised and gel-purified (Bioline ISOLATE II PCR and Gel Kit, United Kingdom), and the recipient and mutant fragments were ligated (Promega T4 ligation kit, United States of America) and transformed by electroporation (XL1 electroporation-competent cells, Agilent Technologies, United States). This enabled the insertion of the mutant fragments into the recipient vector.

The plasmids used in this study are listed in Supplementary Table S1.

### Cell culture

Human embryonic kidney 293T (HEK293T) cells were grown in Dulbecco’s modified Eagle’s medium (DMEM) (Sigma-Aldrich, United Kingdom) supplemented with 10% (v/v) heat-inactivated fetal bovine serum (FBS) (Life Technologies, United States) and 1% (v/v) 2 mM L-glutamine (Sigma-Aldrich, United Kingdom). Cells were incubated at 37°C in 5% CO_2_. Versene solution (Life Technologies, United States) was used for non-enzymatic cell dissociation.

### Transient transfection

HEK293T cells were transfected with either wild-type *CACNA1F* or mutant constructs using FuGENE HD Transfection Reagent (Promega Ltd., United States) in Opti-MEM media (Life Technologies, United States) in a 1 α_1.4_: 0.6 β_3_: 0.8 α_2_δ ratio. A pEGFP plasmid was used for detection (1 α: 0.2 pEGFP). Transfected cells were incubated at 37°C in 5% CO_2_, and for electrophysiological analysis, the temperature was reduced to 30°C after 6–8 h at 37°C to ensure stable calcium ion currents (Koschak et al., 2003). The transfection medium was replaced with culture medium 24 h post-transfection and incubated for a further 24 h.

A β_3_ subunit was used instead of the retina-specific β_2_ for better expression, and higher currents are recorded with no changes in gating properties (Koschak et al., 2003; Hoda et al., 2005).

### Inhibitor treatment

Each drug was added directly to the cell culture media for the stated time prior to harvesting for patch-clamp or Western blot analysis.

A measure of 20 μg/mL cycloheximide (CHX) was added to the cell culture media at different time points prior to harvesting for Western blot analysis.

The inhibitors used in this study are listed in Supplementary Table S2.

### Whole-cell patch-clamp analysis

Transfected HEK293T cells expressing Ca_v_1.4 were dissociated and seeded on poly-L-lysine (0.05%)-coated coverslips 24 h prior to analysis and maintained at 30°C in 5% CO_2_. For electrophysiology recording, the coverslips were placed in extracellular buffer supplemented with calcium ions as the permeating ion (15 mM CaCl_2_, 150 mM choline-Cl, 10 mM HEPES, and 1 mM MgCl_2_, adjusted to pH 7.3 with 1 M CsOH). Borosilicate glass capillaries GC100F-10 (Harvard Apparatus, United Kingdom) were pulled with a Model P-97 pipette puller (Sutter Instrument Co., United States of America) to a resistance of 2–4 MΩ (unpolished). The pipettes were filled with intracellular buffer (5 mM EGTA, 140 mM *N*-methyl-*D*-glucamine, 2 mM MgCl_2_, 10 mM HEPES, and 2 mM Mg-ATP, adjusted to pH 7.3 with 1 M methanesulfonic acid). The osmolarity of all buffers was adjusted to 290–310 using D-mannitol (Osmomat 3000: Gonotec, Germany). Only cells co-expressing GFP were patched. Cells were held at −80 mV, and currents were evoked by 5 ms depolarization from −80 to 80 mV using an online leak subtraction protocol (P/4). Three separate traces were recorded and averaged per cell. No significant difference in cell capacitance (average capacitance being 12 pF) was observed, negating the need to represent the data as current density.

Conventional whole-cell recordings were performed using a MultiClamp 700A amplifier, Digidata 1440A digitizer, and pCLAMP v10 (Molecular Devices, CA, United States of America). Recordings were digitized at 20 KHz and filtered at 10 KHz. Leak-subtracted currents were analyzed on Clampfit software v11.0.3.

### SDS-PAGE and Western blotting

Membrane protein lysates were harvested from 60-mm dishes following the instructions in the Mem-PER Plus Membrane Protein Extraction Kit (Thermo Fisher, United Kingdom), supplemented with proteasome inhibitor cocktail (Sigma-Aldrich, United Kingdom). Protein lysates were prepared with 2x Laemmli sample buffer (1: 1) (Sigma-Aldrich, United Kingdom) supplemented with β-mercaptoethanol (Sigma-Aldrich, United Kingdom). These were loaded on a 4%–20% Mini-PROTEAN TGX Stain-Free Gel (Bio-Rad, United Kingdom). Gels were transferred onto a nitrocellulose membrane (LI-COR Biosciences, United Kingdom) by wet transfer at 350 mA for 90 min at 4°C. The membrane was blocked in 5% milk in TBS-T at room temperature for 1 h before adding the primary antibody in 2% milk TBS-T for 1 h at room temperature. The membrane was washed three times in TBS-T and incubated with the secondary antibody in 2% milk TBS-T for 1 h at room temperature. After three washes in TBS-T, the membrane was imaged using the LI-COR Odyssey CLx system, and LI-COR Image Studio v5.0 was used to analyze the image. The antibodies used in this study are listed in Supplementary Tables S3, S4.

### Data analysis

Quantitative data for Western blots were combined from at least three independent experiments and expressed as mean ± standard error of the mean (S.E.M). A minimum of three biological replicates were used for statistical analysis. Patch-clamp data were acquired from at least three independent experiments and expressed as mean ± standard error of the mean (S.E.M), with each individual cell recording repeated three times and averaged. Statistical significance was determined by Student’s t-test or one-way ANOVA with the Bonferroni correction, with a statistically significant difference defined at *p* < 0.05. The Student’s t-test was used when the limitation of replicates prevented the use of the Kruskal–Wallis test. All data were analyzed and plotted on GraphPad Prism v8.0 (GraphPad, La Jolla, United States).

## Results

### Selection of *CACNA1F* variants for functional analysis

We selected 10 *CACNA1F* VUS from local patients via a putative CSNB2 diagnosis (Table 1). As it was shown previously that the connecting loops are the most frequently substituted regions in the Ca_v_1 family (Sadeh et al., 2021), we selected two variants in these loops: p.D119Y (identified in a male patient with an electronegative electroretinogram (ERG)) and p.E797V (identified in two unrelated families (Hove et al., 2016)). We further chose four variants in the pore-forming domains: p.R290C, p.G674S, p.D1097N, and p.N1434S. These pore variants are functionally constrained and likely to dysregulate Ca^2+^ influx (Sadeh et al., 2021). All four Ca_v_1.4 pore variants are novel and were present in male patients that had ERGs consistent with diagnosis of CSNB2; p. R290C was present in four family members and p.N1434S was present in two cousins, all affected with CSNB2 (Table 1).

**TABLE 1:** Clinical details and properties of the 10 novel *CACNA1F* variants of unknown significance (VUS) identified at the Manchester Genomics Diagnostic Laboratory in patients with congenital stationary night blindness (CSNB). The degree of residue conservation is calculated using Clustal Omega (Sievers et al., 2011) on three paralogues and 20 orthologues. CACNA1F-vp (Sallah et al., 2020) prediction of pathogenicity.

VUS (cDNA, protein)	Clinical detail	Structural location	Conservation	CACNA1F-vp	Reference
c.355G>T, p. (Asp119Tyr)	Consistent with CSNB2	R1S1 EL	High	Pathogenic	
	Attenuated light adapted and attenuated B waves on light-adapted ERG				
	No family history				
c.868C>T, p. (Arg290Cys)	VA (LogMAR) 0.92/0.76	R1S5 EL	High	Benign	10.1016/j.ophtha.2017.02.005
	Refractive correction (diopter) −7/-8				
	Horizontal nystagmus				
	Two siblings				
	Consistent with CSNB2				
	Affected cousin				
	Attenuated light-adapted and attenuated B waves on light-adapted ERG				
	Affected cousin				
	Electronegative ERG				
c.2020G>A, p. (Gly674Ser)	Consistent with CSNB2	R2S5	High	Pathogenic	
c.2390 A>T, p. (Glu797Val)	Unknown	R2S6 CL	Low	Benign	DOI:10.1167/iovs.16-19445
c.3289G>A, p. (Asp1097Asn)	VA (LogMAR) 0.82/0.64	R3S5 EL	High	Pathogenic	10.1016/j.ophtha.2017.02.005
	Refractive correction (diopter) −17/-16D				
	Cone dysfunction with abnormal B wave on dark-adapted bright flash ERG				
	No family history				
c.4301 A>G, p. (Asn1434Ser)	Consistent with CSNB2	R4S6	High	Pathogenic	10.1016/j.ophtha.2017.02.005
	VA (LogMAR) 0.70/0.77				
	Horizontal nystagmus				
	Male cousins				
	ERG consistent with CSNB2				
c.4472C>T, p. (Pro1491Leu)	Suggestive CSNB2	CTD	High	Pathogenic	DOI:10.1016/j.ajhg.2016.12.003, DOI:10.1038/s41586-020-2434-2
	VA (LogMAR) 0.26/0.34				
	Refractive correction (diopter) +6				
	Poor cone responses with attenuated B waves in dark ERG				
c.4480G>A, p. (Gly1494Arg)	Unknown	CTD	High	Pathogenic	DOI:10.1016/j.preteyeres.2014.09.001
c.4518G>T, p. (Lys1506Asn)	Consistent with CSNB2	CTD	High	Pathogenic	
c.4594C>T, p. (Arg1532Trp)	Four affected in the family; X-linked (but no co-segregation)	CTD	High	Pathogenic	DOI:10.1167/iovs.16-19445
	Nystagmus				
	Normal fundus appearance				
	ERG suggests combined cone and rod problem				

Structural location: CL, cytoplasmic loop; CTD, carboxyl-tail domain; EL, extracellular loop; R, repeat; S, segment. Clinical details: ERG, electroretinogram; CSNB2, incomplete congenital stationary night blindness type 2; VA, visual acuity.

The carboxyl-tail domain (CTD) of Ca_v_1.4 is structurally unresolved, thus preventing accurate predictions of the functional consequence of variation in this region. To address this, we characterized four *CACNA1F* VUS in the CTD (p.P1491L, p.G1494R, p.K1506N, and p.R1532W) that were predicted as pathogenic by CACNA1F-vp (Sallah et al., 2020) (Table 1). We and other researchers have reported p.P1491L in patients with ERGs typical of CSNB2 (Carss et al., 2017; Turro et al., 2020) and identified the previously reported p.R1532W variant in multiple affected family members (Hove et al., 2016).

**FIGURE 1:**
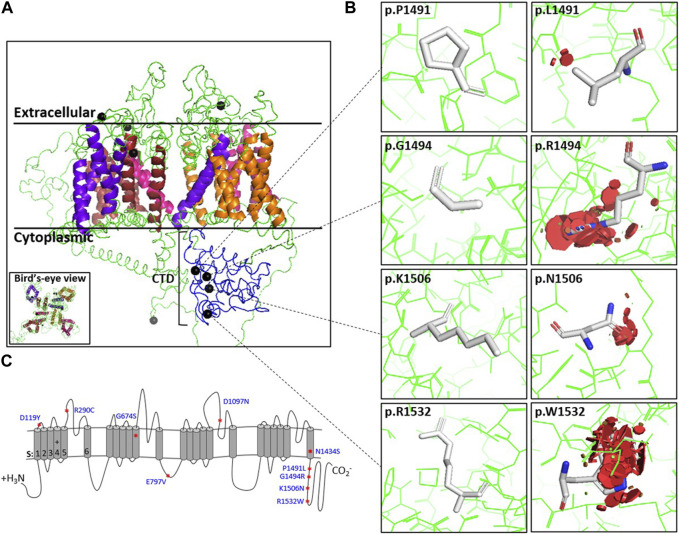
Position and steric clashes of Ca_v_1.4 missense variants. The locations of the Ca_v_1.4 variants from this study are shown on a homology model of Ca_v_1.4. **(A)** Cross-section of the Ca_v_1.4 homology model (extracellular, transmembrane, and cytoplasmic regions indicated). Helices are colored for each repeat. Blue indicates the carboxyl-tail. The black spheres indicate the position of the Ca_v_1.4 variant. **(B)** Modeling of the Ca_v_1.4 variants of uncertain significance (VUS) (shown as PyMOL images) predicted that they are likely to cause protein hindrance by inducing steric clashes. The red discs demonstrate the predicted degree of steric clashes between the mutated residue and surrounding residues. **(C)** Picture representation of Ca_v_1.4’s linear structure. Additional variant PyMOL images are provided in Supplementary Figure S1.

We modeled all 10 mutations on an *in silico* homology model of Ca_v_1.4. The model was used to predict the physiochemical consequences of each missense variant on steric clashes due to charge, polarity, or bond disturbances (Figure 1; Supplementary Figure S1). Although all *in silico* modeling techniques have their limitations, our modeling and informatics analyses predict that all 10 variants would likely result in steric clashes that are likely to cause instability to the variant channel (Table 1).

### *CACNA1F* missense mutations reduce channel stability, expression, and function

We next sought to confirm the pathogenic predictions of the 10 variants on channel function by whole-cell patch-clamp analysis, quantifying channel current as a measure of channel function. The global expression and protein stability of the 10 novel variants were determined by Western blotting and cycloheximide (CHX) chase assay, respectively.

**FIGURE 2:**
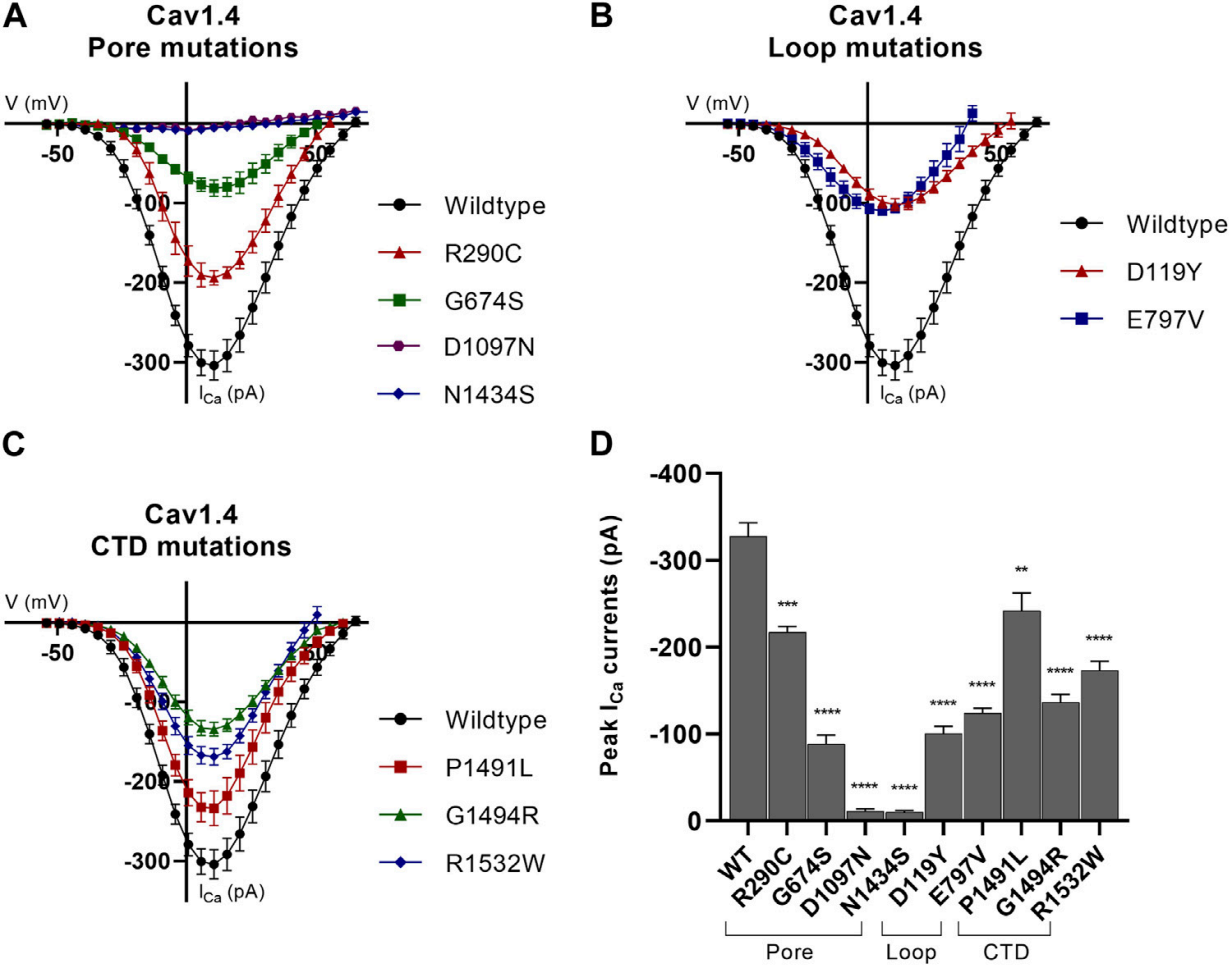
Whole-cell ion currents of Ca_v_1.4 variants. I/V plots for **(A)** wild-type Ca_v_1.4 , pore-forming domains, and associated loop mutations, **(B)** wild-type Ca_v_1.4 and loop (excluding the pore-forming S5–S6 loop) mutations, and **(C)** wild-type Ca_v_1.4 and carboxyl-tail domain (CTD) mutations. The CTD variant, p.K1506N, is omitted from this figure as no statistical significance from the wild type was identified (data are presented in Supplementary Table S5). The wild-type Ca_v_1.4 I_Ca_ activates at ∼−45 mV and reverses at ∼45 mV. All nine variants significantly reduce the peak I_Ca_ compared to the wild type. Representative raw traces are presented in Supplementary Figure S2. Currents were evoked by 5 ms depolarization from −80 to 80 mV with P/4 leak subtraction. The wild-type Ca_v_1.4 traces are an accumulation from multiple experiments (*n* = 30). A minimum of *n* = 10 traces per variant with three separate recordings were taken per cell and averaged. Error bars represent mean ± S.E.M. **(D)** Peak I_Ca_ currents for wild-type and mutant Ca_v_1.4 channels. Peak current differences between wild type and mutant were analyzed by one-way ANOVA with the Bonferroni correction. *p < 0.05, **p < 0.01, and ***p < 0.001 indicate the degrees of significance (*p*-values are provided in Supplementary Table S5). Error bars represent mean ± S.E.M. Pore mutations, S5–S6 pore-forming membrane segments and connecting loop mutations; loop mutations, mutations in loops excluding pore-forming S5–S6 loop; CTD mutations, mutations in the carboxyl-tail domain; WT, wild type.

We identified a significant reduction in expressed Ca^2+^ current (I_Ca_) for all but one variant. There was a 62% mean (range 26%–96%) reduction in peak I_Ca_ compared to the human wild-type Ca_v_1.4 channel (Figure 2; Supplementary Table S5). There was also a significant reduction in global protein expression for nine of the 10 novel variants, with a mean reduction of 34% (range 12%–51%) compared to the wild-type (Supplementary Figure S3; Supplementary Table S5).

**FIGURE 3:**
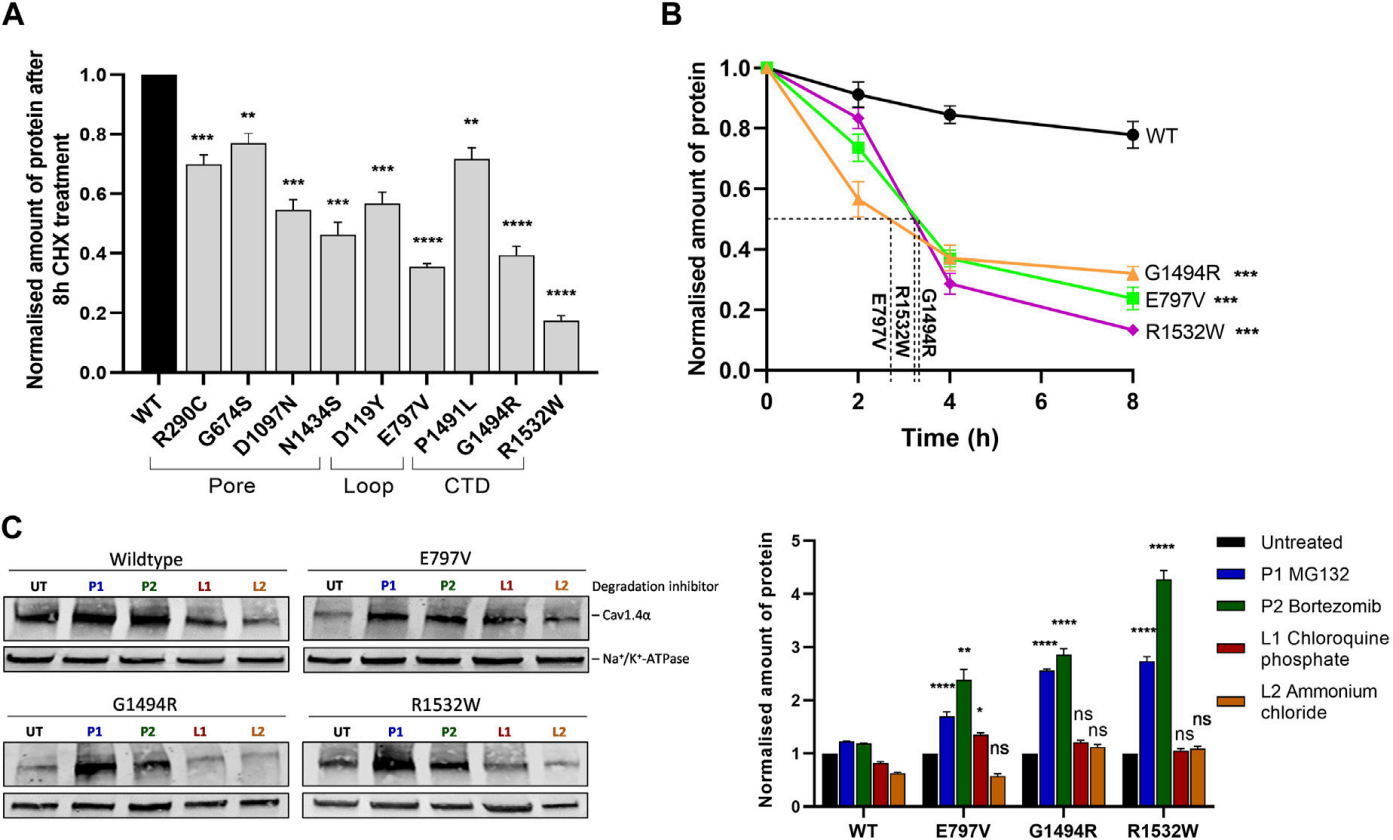
Ca_v_1.4 variants reduce protein stability and are degraded by the proteasome. **(A)** Quantified Western blotting data from cells expressing Ca_v_1.4 variants treated with 20 μg/mL cycloheximide (CHX) for 8 h. Error bars represent mean ± S.E.M of three independent experiments. Wild-type expression is set at 1. *p < 0.05, **p < 0.01, and ***p < 0.001 indicate the degrees of significance for the remaining protein between each variant and the wild-type protein analyzed by a one-way, unpaired Student’s t-test (*p*-values are provided in Supplementary Table S5). A representative Western blot is shown in Supplementary Figure S4A. **(B)** Quantified Western blot data of the three least stable mutations (p.E797V, p.G1494R, and p.R1532W) after different CHX treatment times (0, 2, 4, and 8 h) with the protein half-life shown. Error bars represent mean ± S.E.M of three independent experiments. The amount of protein is shown relative to untreated (0 h) expression, which is set at 1. A one-way, unpaired Student’s t-test analysis compares the amount of mutant protein remaining after 8 h relative to the wild type. Representative Western blots are shown in Supplementary Figure S4B. **(C)** Representative Western blot **(left)** and quantified expression **(right)** of wild-type Ca_v_1.4 and the three least stable proteins treated with proteasome or lysosome inhibitors for 6 h. Error bars represent mean ± S.E.M of three independent experiments. The amount of Ca_v_1.4 protein (220 kDa) was normalized to the loading control (Na^+^/K^+^-ATPase, 110 kDa) and is relative to the untreated controls that are set at 1. *p < 0.05, **p < 0.01, and ***p < 0.001 indicate the degrees of significance between treated and untreated cells using a one-way, unpaired Student’s t-test. WT, wild type; UT, untreated; P1 (bortezomib, 10 nM) and P2 (MG132, 20 μM), proteasome inhibitors; L1 (chloroquine phosphate, 50 μM) and L2 (ammonium chloride, 50 mM), lysosome inhibitors.

Protein turnover was significantly increased for nine variant proteins relative to the wild-type. On average, just over half (52%) of the variant protein remained after 8 h CHX chase compared to the wild-type variant protein (range 17%–77%) (Figure 3A, Supplementary Figure S4, Supplementary Table S5). The rate of protein turnover for the Ca_v_1.4 pore variant p.E797V and two Ca_v_1.4 CTD variants (p.G1494R and p.R1532W) increased compared to that of the wild-type protein (Figure 3B; Supplementary Figure S4). There was an average 3 h half-life for these three mutant proteins, whereas 75% of the wild-type protein remained till the end of the chase (Figure 3B).

The increased turnover in mutant proteins is consistent with the homology model’s prediction of protein instability due to steric clashes (Figure 1; Supplementary Figure S1). This protein instability was observed in all the Ca_v_1.4 mutations studied.

For three variants, the functional data did not support the informatics predictions. The Ca_v_1.4 variants p.R290C and p.E797V, neither of which is found in gnomAD, were hypomorphic on functional analysis but were classified as benign variants by CACNA1F-vp. Additionally, the Ca_v_1.4 CTD variant p.K1506N, which was predicted to be pathogenic by CACNA1F-vp, had currents, expression, and stability comparable to the wild-type channel (Supplementary Table S4).

### Mutant Cav1.4α proteins are degraded by the proteasome

We used the three least stable novel variants (p.E797V, p.G1494R, and p.R1532W) to identify the route of degradation for Ca_v_1.4α variants. Non-native proteins can be degraded *via* proteasomes or lysosomes through the ubiquitin-proteasome pathway or autophagy, respectively (Levine and Klionsky, 2004; Collins and Goldberg, 2017). We inhibited each pathway using either the proteasome inhibitors (PIs) (bortezomib (BTZ) and MG132) or the lysosome inhibitors (LIs) (chloroquine phosphate and ammonium chloride) for 6 h. BTZ and MG132 are highly selective PIs that inhibit the proteolytic activity of the 26S proteasome complex (Han et al., 2009; Fricker, 2020), whereas chloroquine phosphate and ammonium chloride are weak bases that inhibit autophagy by changing the lysosomal pH or by inhibiting phagosome–lysosome fusion, respectively (Hart and Young, 1991; Redmann et al., 2017). We found that the ubiquitin-proteasome degradation pathway is the major route of degradation for the Ca_v_1.4α variants investigated. After treatment with PIs, the amount of mutant Ca_v_1.4α proteins detected increased by 2– 4-fold relative to untreated cells (Figure 3C). This increase was highly significant. In contrast, ammonium chloride made no significant difference to the detectable mutant Ca_v_1.4α proteins, and chloroquine phosphate only marginally increased the expression of one mutant protein (p.E797V) (Figure 3C).

### Proteasome inhibitors restore expression and function of Ca_v_1.4 mutant channels

**FIGURE 4:**
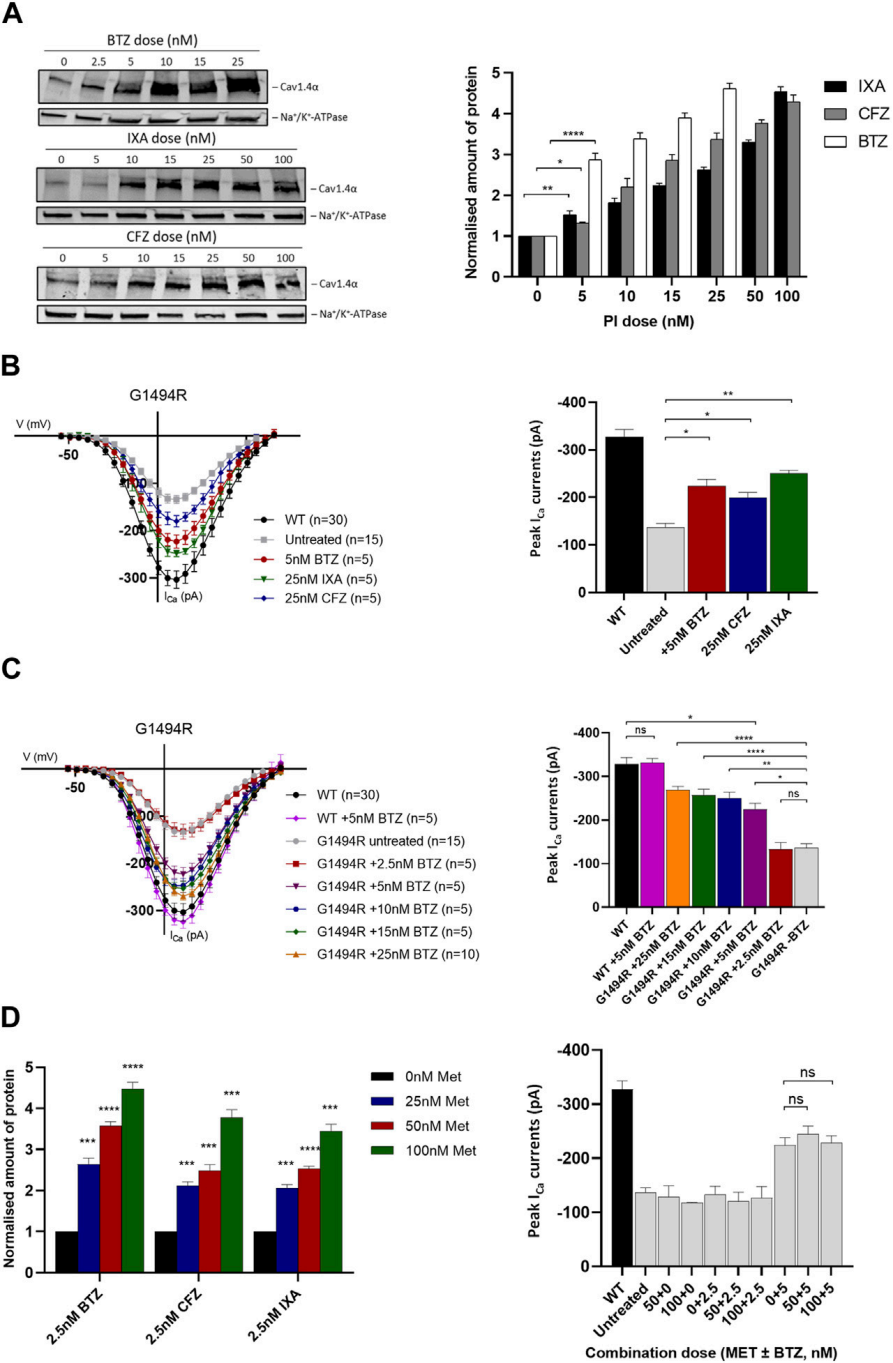
Ca_v_1.4 protein expression and channel function following treatment with proteasome inhibitors and metformin. **(A)** Dose–response relationship of Ca_v_1.4 p.G1494R and proteasome inhibitors (PIs) after 6 h treatment. Representative Western blot **(left)** and quantified plot **(right)** of p.G1494R treated with increasing doses of each PI. Error bars represent mean ± S.E.M of three independent experiments, and statistical significance is measured by a one-way, unpaired Student’s t-test. The Ca_v_1.4 protein (220 kDa) was normalized to the loading control (Na^+^/K^+^-ATPase, 110 kDa) and is relative to the untreated control set at 1. **(B)** I/V plots of Ca_v_1.4 p.G1494R treated with different PIs for 6 h **(left)** and the peak I_Ca_ **(right)**. Error bars represent mean ± S.E.M., and statistical significance is analyzed by a one-way, unpaired Student’s t-test. **(C)** I/V plots of I_Ca_ for Ca_v_1.4 p.G1494R treated with increasing concentrations of bortezomib (BTZ) for 6 h **(left)** and the peak I_Ca_ **(right)**. Error bars represent mean ± S.E.M., and statistical significance is analyzed by a one-way, unpaired Student’s t-test. **(D)** Quantified Western blot data of Ca_v_1.4 p.G1494R treated with each PI combined with different concentrations of metformin for 6 h **(left)**. Error bars represent mean ± S.E.M of three independent experiments. A one-way, unpaired Student’s t-test analysis was used to compare the relative amount of protein per drug combination relative to metformin alone. A representative Western blot is shown in Supplementary Figure S5A. Peak I_Ca_ **(right)** of Ca_v_1.4 p.G1494R treated with different combinations of metformin and BTZ. I/V plots are shown in Supplementary Figure S5B. For I/V plots, the currents were evoked by 5 ms depolarization from −80 to 80 mV with P/4 leak subtraction. The wild-type Ca_v_1.4 traces are an accumulation from multiple experiments (*n* = 30). Each cell trace was recorded three times, and an average was taken. Error bars represent mean ± S.E.M. The peak I_Ca_ are normalized to the wild type. *p < 0.05, **p < 0.01, and ***p < 0.001 indicate the degrees of significance between groups analyzed by a one-way, unpaired Student’s t-test. Met, metformin; BTZ, bortezomib; CFZ, carfilzomib; IXA, ixazomib; WT, wild type.

Having determined that the proteasome is the most likely route for degradation of mutant Cav1.4α proteins, we postulated that inhibiting this pathway may be therapeutically beneficial by increasing the levels of mutant channel expression/function. We tested three clinically approved PIs: BTZ, carfilzomib (CFZ), and ixazomib (IXA). We first tested their effects on Ca_v_1.4α p.G1494R since this mutant channel had significantly reduced expression and was particularly unstable. All three PIs had comparable toxic effects *in vitro*, and we selected a treatment time of 6 h as >50% of the cells were still viable after this time (data not shown). All three inhibitors significantly increased the amount of detectable protein after 6 h treatments, with the highest doses of PIs resulting in a 5-fold increase in protein expression relative to untreated samples (Figure 4A). Bortezomib was the most potent PI, with ×5 as much as CFZ (25 nM compared to 5 nM) and ×10 as much as IXA (50 nM compared to 5 nM) being required to achieve a similar increase in protein expression.

Using whole-cell peak I_Ca_ as a measure of channel function, we were able to significantly increase the cellular function of Ca_v_1.4 p.G1494R in cells treated with each PI compared to untreated ones (Figure 4B). BTZ was the most potent, while higher concentrations of CFZ and IXA were required to achieve comparable increases (Figure 4B). As 2.5 nM BTZ had no significant effect on the peak I_Ca_ or protein expression compared to untreated cells (Figure 4C), we titrated the concentration of BTZ and determined 5 nM as the threshold to achieve a significant increase in function and expression whilst maintaining minimal toxicity to cells expressing Ca_v_1.4 p.G1494R (Figure 4C). Higher concentrations of BTZ further increased mutant channel I_Ca_, achieving >80% wild type with 25 nM BTZ.

Given the toxicity of the PIs tested, we tried to reduce the PI concentration required to achieve a significant increase in expressed I_Ca_ by combining each with metformin (Met). Metformin is not only approved for the treatment of type 2 diabetes but also acts to suppress the expression of GRP78-dependent autophagy and enhance the pharmacological effects of BTZ (Jagannathan et al., 2015). We combined a low concentration of each PI that had no effect on expressed I_Ca_ (2.5 nM) with increasing concentrations of Met and measured the channel expression and function of the p.G1494R mutant after 6 h. Metformin had no effect on either protein expression or peak I_Ca_ on its own at any of the concentrations tested (Figure 4D; Supplementary Figure S5A). However, in combination with any of the three PIs, it significantly increased mutant p.G1494R Ca_v_1.4α expression, and this combination was Met dose-dependent (Figure 4D, Supplementary Figure S5A). Despite the increase in channel expression, no Met–PI combination had any effect on mutant p.G1494R Ca_v_1.4 peak I_Ca_ (Figure 4D; Supplementary Figure S5B).

**FIGURE 5:**
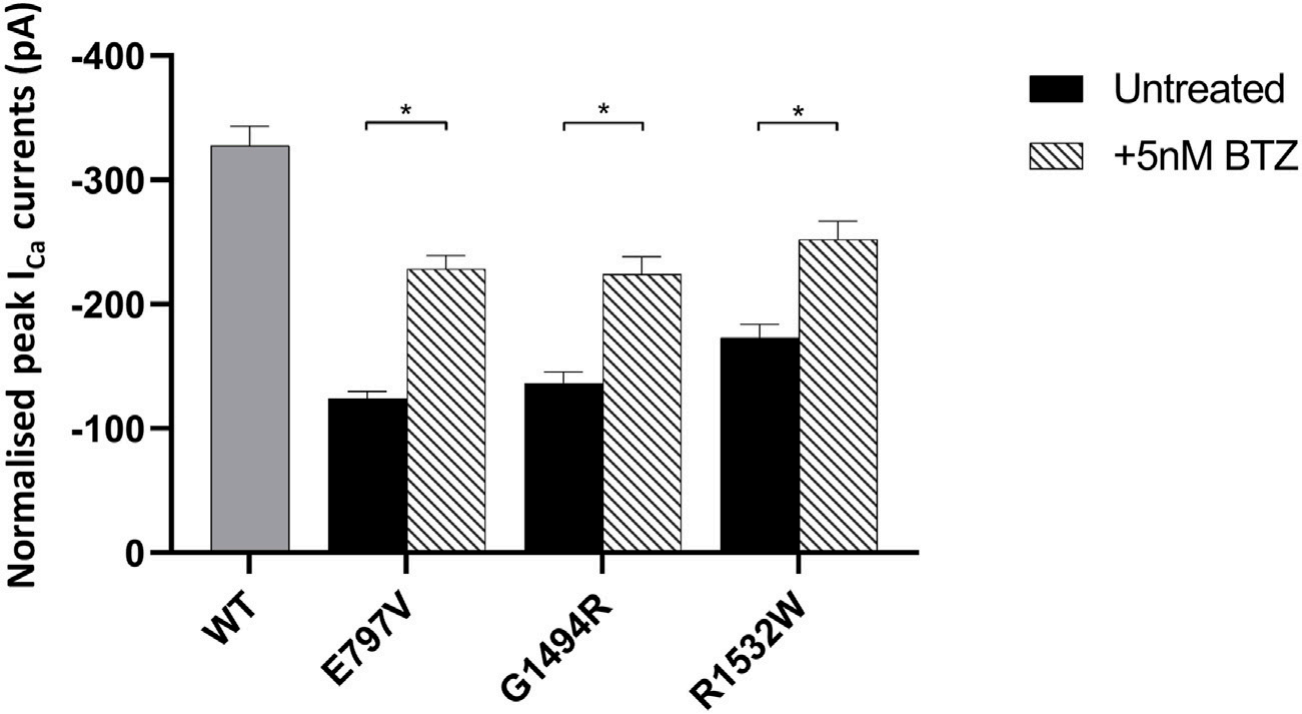
Effect of bortezomib on the function of mutant Ca_v_1.4 channels. Bar chart plot of the peak I_Ca_ for wild-type and mutant Ca_v_1.4 treated with 5 nM bortezomib (BTZ) for 6 h compared to untreated controls. BTZ had no effect on wild type (data not shown). Error bars represent mean ± S.E.M. *p < 0.05, **p < 0.01, and ***p < 0.001 indicate the degrees of significance between treated and untreated channels analyzed by a one-way, unpaired Student’s t-test. WT, wild type; BTZ, bortezomib.

Having discounted the combined use of Met with a PI, we next investigated the ability of 5 nM BTZ to increase expressed peak I_Ca_ for the two least stable Ca_v_1.4 mutant proteins (p.E797V and p.R1532W) (Figure 5; Supplementary Figure S6). BTZ significantly increased the peak current of both mutants, with a mean increase in peak I_Ca_ greater than 70% relative to each wild type after a 6-h treatment (Figure 5).

## Discussion

The Ca_v_1 protein family is evolutionarily conserved (Catterall et al., 2005; Zamponi et al., 2015). The encoded genes are mutated in a range of rare disorders inherited as autosomal dominant, recessive, and X-linked traits affecting a wide range of organ systems reflecting their expression patterns. Pathogenic variants in *CACNA1F*, which is highly expressed in the retina, cause X-linked CSNB2, a rare disorder causing visual disability in males. In this context, the interpretation of missense variant pathogenicity across the entire Ca_v_1 family remains challenging (Brunklaus et al., 2014; Heyne et al., 2020; Indelicato and Boesch, 2021; Sadeh et al., 2021) and can delay diagnosis and treatment. To this end, we and other researchers have developed informatics tools, including CACNA1F-vp and funNCIon (Heyne et al., 2020; Sallah et al., 2020), and in this experimental follow-up study, have undertaken functional studies to examine the efficacy of these tools.

We examined 10 missense variants that are located throughout the Ca_v_1.4α protein, including those within connecting loops, pores, and C-terminal tail. Nine of the 10 variants showed a dramatic reduction in whole-cell Ca^2+^ current (I_Ca_) compared to cells expressing the wild-type channel, associated with low levels of expression and an increased rate of protein turnover. The increased turnover of mutant proteins is consistent with the predicted impact of steric clashes from homology modeling and suggests a common pathological mechanism. Of the parameters examined, there was a correlation between the expression level of the mutant protein compared to the wild-type channel. The four pore mutants were the most highly expressed, all being above 70% of the wild-type channel. Associations between I_Ca_ and protein stability were less clear-cut. The three mutants with the largest decrease in I_Ca_ were all in the pore (p.G674S, p.D1097N, and p.N1434S). Two of the three most stable mutant channels (protein remaining after CHX treatment) were in the pore (p.R290C and p.G674S) along with p.P1491L in the CTD. The p.K1506N CTD mutant channel showed little difference from the wild-type channel, and it is likely that this splice site variant is non-pathogenic.

A wide range of mutations underlies *CACNA1F*-related CSNB2, including both classical LoF variants (nonsense, splice site, and indel) and missense alterations. Our data highlight that pathogenic missense changes in Ca_v_1.4α act through an LoF mechanism and, using three novel variants, that this is consequent upon protein instability and ubiquitin-proteasome degradation. Proteasome inhibition with four different inhibitors resulted in increased protein expression and ion conductance (Figure 3C; Figures 4A–C). This suggests that mutant Cav1.4α is degraded by endoplasmic reticulum-associated protein degradation and prevents trafficking of the mutant protein to the plasma membrane. This has been described for a number of inherited conditions, including the degenerative retinal condition retinitis pigmentosa (RP) (Abriel et al., 2000; Benhorin et al., 2000; Macías-Vidal et al., 2014; Zhang et al., 2019). A drug capable of targeting mutations across a family of proteins and a range of disorders is an attractive property for a therapeutic, as exemplified by the use of flecainide to target voltage-gated sodium channels in the heart (SCN5A) and skeletal muscle (SCN4A) (Ward et al., 1995; Rosenfeld et al., 1997; Galbiati et al., 2000; Zheng et al., 2012). Inhibiting the turnover of mutant proteins has been investigated as a possible therapeutic treatment for dominant negative mutations in caveolin-3 associated with limb-girdle muscular dystrophy (Galbiati et al., 2000). For cystic fibrosis, two general PIs restored deltaF508 CFTR expression in the plasma membrane, although these were immature forms and so of limited therapeutic value (Ward et al., 1995). We found three clinically approved PIs, and all significantly increased the channel density of Ca_v_1.4α mutations, with BTZ being the most potent. This is consistent with other studies using BTZ to increase the gross cellular function of proteins mutated in a range of conditions, including RP, Niemann–Pick disease type C, mitochondrial leukoencephalopathy, and Lynch syndrome (Macías-Vidal et al., 2014; Zhang et al., 2019). There are trials for more than 600 conditions investigating BTZ as a therapy (ClinicalTrials.gov), although its long-term use has been associated with side effects, such as peripheral neurotoxicity and mitotoxicity (Cavaletti et al., 2007; Nowis et al., 2010; Zheng et al., 2012; Kaplan et al., 2017). The BTZ concentrations that are shown to be effective against Ca_v_1.4α mutations are within the range approved for clinical use, suggesting that BTZ proteasomal inhibition also has therapeutic potential for the treatment of CSNB2.

Metformin is a first-line treatment for type 2 diabetes and has been researched for its many other effects, including the management of polycystic ovary syndrome. In model systems, it can alleviate polycystic kidney disease (Pastor-Soler et al., 2022), and for cancer treatment, it can modulate the unfolded protein response resulting from bortezomib treatment (Jagannathan et al., 2015; Granato et al., 2017). In an RP mouse model, metformin was able to stabilize mutant rhodopsin and enhance its trafficking to the rod outer segment. The latter effect appears to be predominant as this resulted in increased photoreceptor death (Athanasiou et al., 2017). Given these reported modes of action, we tested whether metformin, either alone or in combination with PIs, could increase the function of mutant Ca_v_1.4α. Although metformin significantly increased the expression of mutant Ca_v_1.4α higher than that achieved with a PI alone, it had no effect on current density and was possibly inhibitory to the positive effect of bortezomib. This is in contrast to a previous report that metformin downregulated the expression of Ca_v_1.2, although a study observed that metformin suppressed channel current (Wang et al., 2020). The current density of the Nav1.7 channel is reduced by metformin acting through the E3 ubiquitin ligase NEDD4-2 (Deftu et al., 2022).

The functional consequences of putative pathogenic missense variants in the Ca_v_1.4α CTD have not been previously investigated. The CTD is unresolved in the crystal structure of rabbit Ca_v_1.1α from which the Ca_v_1.4α structure is derived. This reduces the confidence in the Ca_v_1.4α homology model, and consequently, the prediction of variant pathogenicity, and most likely underlies the incorrect functional consequence predictions for three of the 10 Ca_v_1.4 variants (p.R290C, p.E797V, and p.K1506N) (Sallah et al., 2020). We show that mutations in this domain have identical consequences to those in connecting, or pore-forming, loops, namely, a significant reduction in whole-cell I_Ca_, which we correlate to a reduced channel expression resulting from protein instability. CACNA1F-vp correctly predicted the pathological functional consequence of three of the four CTD variants tested (p.P1491L, p.G1494R, and p.R1532W), all of which significantly reduced I_Ca_ of the expressed channel variant. Interestingly, the fourth CTD variant (p.K1506N) that was functionally comparable to wild type lies at the intron–exon boundary with exon 38 and is predicted to alter splicing. Two previously reported pathogenic variants also lie at the same exon–intron boundary: c.4519-1G>A and c.4518 + 2T>A (Rosenfeld et al., 1997; Galbiati et al., 2000).

Like previous electrophysiological studies on Ca_v_1.4, we used heterologous expression in HEK293 cells due to their low expression of native channels and reproducible phenotype observed in mouse models and induced pluripotent stem cells (Burtscher et al., 2014; Hu et al., 2018; Koschak et al., 2021). Future studies should scrutinize the mutant channel physiology in more detail by quantifying changes in gating and deactivation kinetics. Ideally, these experiments should be performed using a more physiological cell line to confirm the pathogenic mechanism of altered protein expression. Quantification of Ca_v_1.4α channel expression on the cell surface would have been beneficial in showing how it changed following proteasomal inhibition. This proved impossible and is consistent with what other laboratories have experienced. Therefore, we relied on the gold-standard whole-cell patch-clamp electrophysiology in combination with semi-quantitative Western blotting as a measure of how ion conductance correlates with channel expression and function.

Being able to provide a molecular diagnosis is important to patients, their families, and their doctors as it confirms the cause of the condition and allows appropriate treatment, support, and counseling. In this study, we empirically test novel *CACNA1F* variants and show that bioinformatics prediction of pathogenicity was 70% accurate. This and future studies are important for refining and improving future prediction algorithms. We show that all *CACNA1F* variants with a functional consequence act via a LoF mechanism, irrespective of the variant’s location in the subunit. The mutant subunits were degraded by the proteasome, and inhibition of this pathway by clinically approved drugs increased the cellular function of the mutant channels. This finding suggests that the use of proteasomal inhibitors may be useful therapeutic drugs for the treatment of CSNB2.

## Data Availability

The original contributions presented in the study are included in the article/Supplementary material; further inquiries can be directed to the corresponding author.
