# Supplementary material for: Cav1.4 congenital stationary night blindness is associated with an increased rate of proteasomal degradation
Source: Front Cell Dev Biol. 2023 May 3;11:1161548. doi: 10.3389/fcell.2023.1161548 (PMC10188973; doi:10.3389/fcell.2023.1161548)
Supplement: Supplementary file 1 [file DataSheet1.PDF]

SUPPLEMENTARY MATERIALS

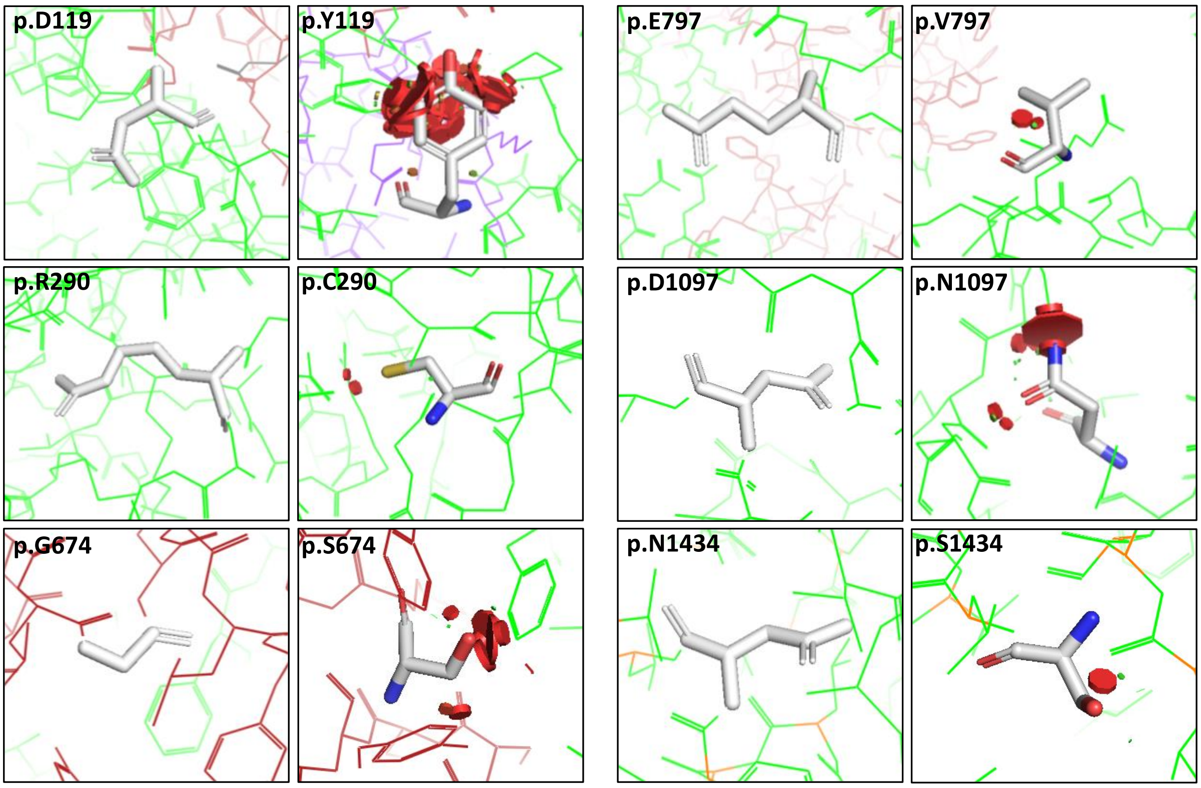

Fig. S1. Modelling of steric clashes in Ca$_v$1.4 mutant channels.

Ca$_v$1.4 homology model showing predicted steric clashes of Ca$_v$1.4 variants of uncertain significance (VUS). PyMol images showing wild type or substituted amino acid residue for Ca$_v$1.4 mutations are shown. Red discs show predicted degree of steric clashes between the mutated residue and surrounding residues.

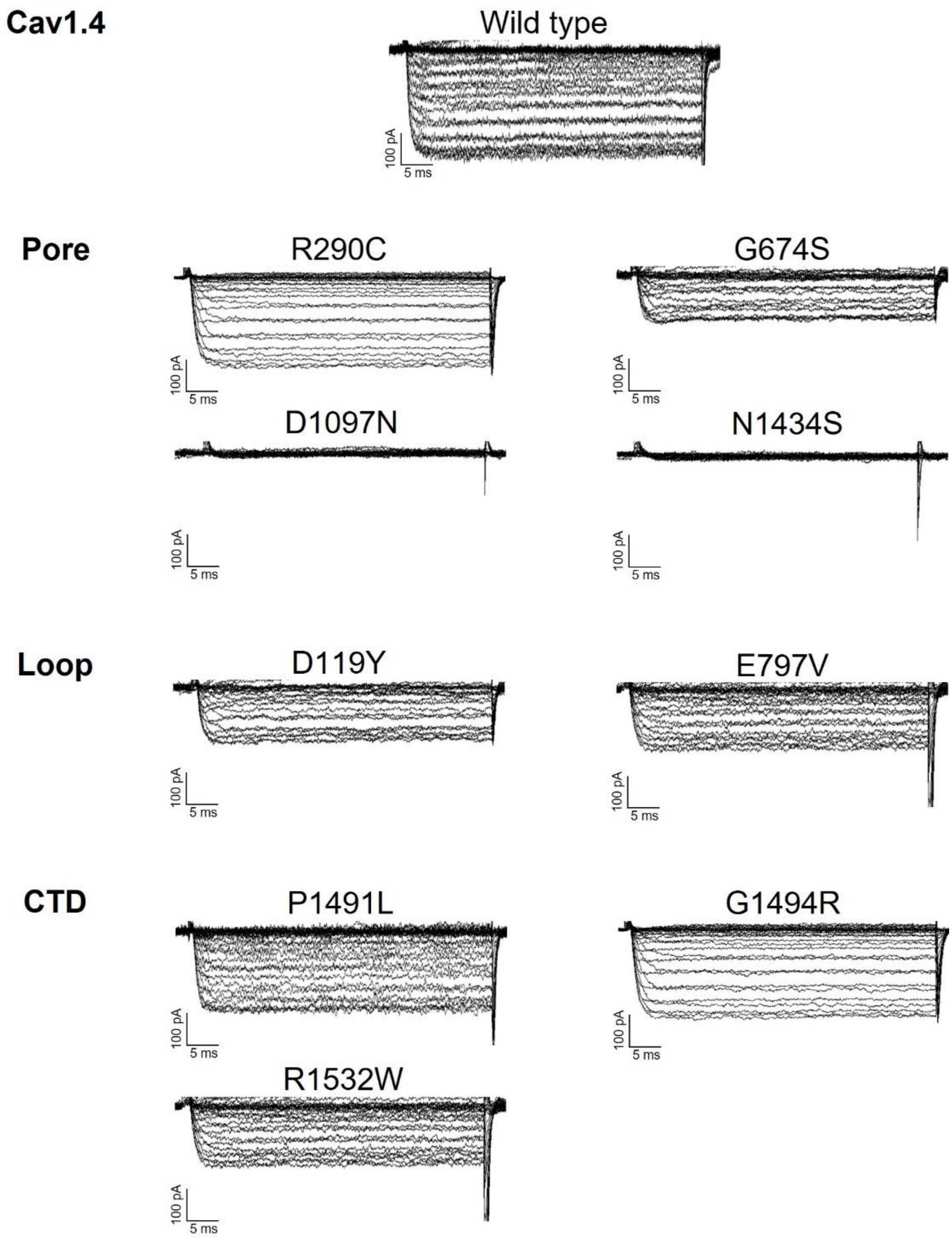

Fig. S2. Raw traces of whole-cell ion currents of Ca_v1.4 variants.

Raw traces of I_{Ca} for Ca_v1.4 mutations. Mutant channel reduced I_{Ca} currents compared to the wild type. Currents were evoked by 5 ms depolarisation from -80 to 80 mV with P/4 leak subtraction. WT, wild type; CTD, carboxyl-tail domain.

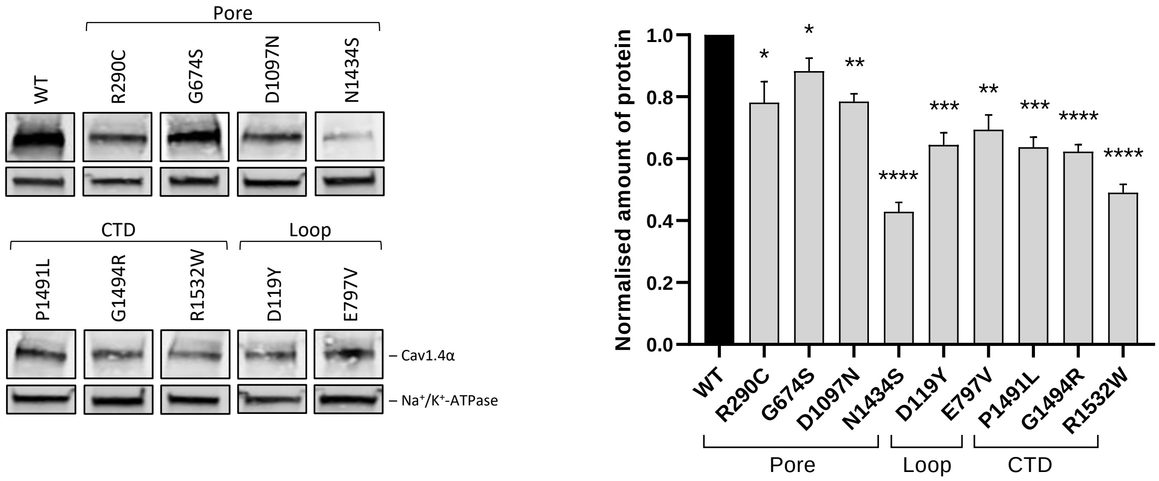

Fig. S3. Effect of Ca_v1.4 mutation on protein expression.

Representative western blots of global Ca_v1.4 protein expression for wild type and mutant channels **(left)** and quantified plot **(right)**. Blot is presented as classes of mutation (pore; loop; CTD (carboxyl-tail domain)). The amount of Ca_v1.4 protein (220 kDa) was normalised to loading control (Na^+/K^+-ATPase, 110 kDa) and is relative to wild type expression. Wild type expression is set as 1. Error bars represent mean ± S.E.M of three independent experiments. *P < 0.05, **P < 0.01, ***P < 0.001 indicates degree of significance analysed by a one-way, unpaired Student’s t-test and p values are in Table S1. WT, wild type; CTD, carboxyl-tail domain.

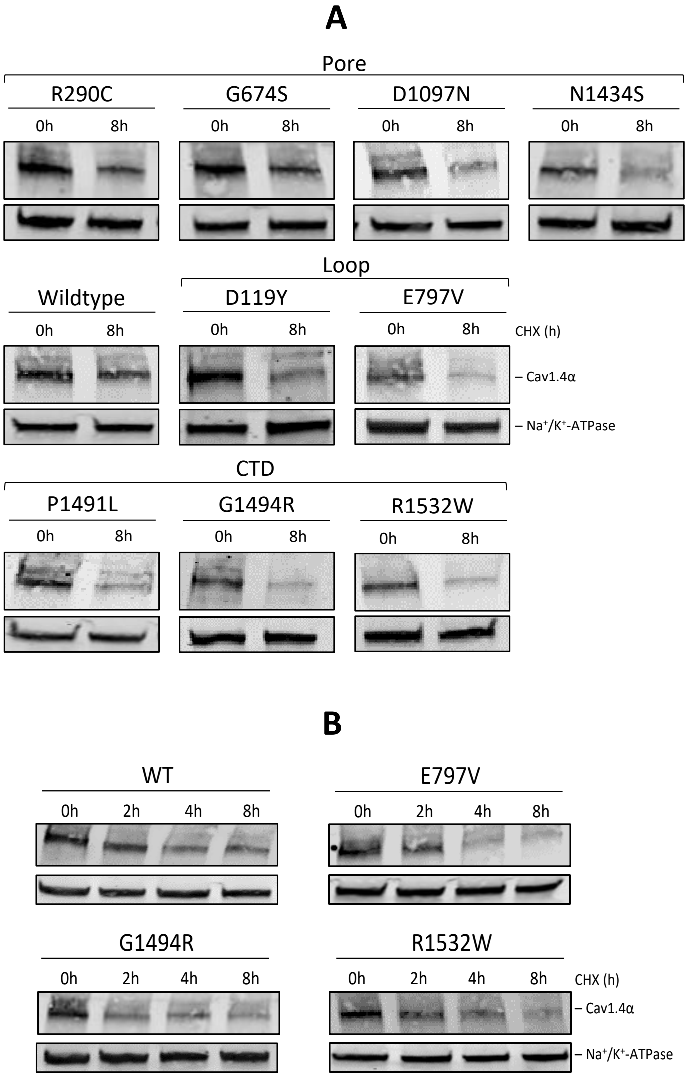

Fig. S4. Protein stability of Ca$_v$1.4 mutations.

(A) Representative western blots of Ca$_v$1.4 proteins treated with 20 µg/ml cycloheximide (CHX) for 8h. Blots are presented as classes of mutations (pore; loop; CTD (carboxyl-tail domain)). **(B)** Representative western blots of the three least stable mutations (p.E797V, p.G1494R, p.R1532W) after different cycloheximide (CHX) treatment times (0, 2, 4, 8h). The Ca$_v$1.4 (220 kDa) and loading control (Na^+/K^+-ATPase, 110 kDa) immunoblot bands are indicated.

A

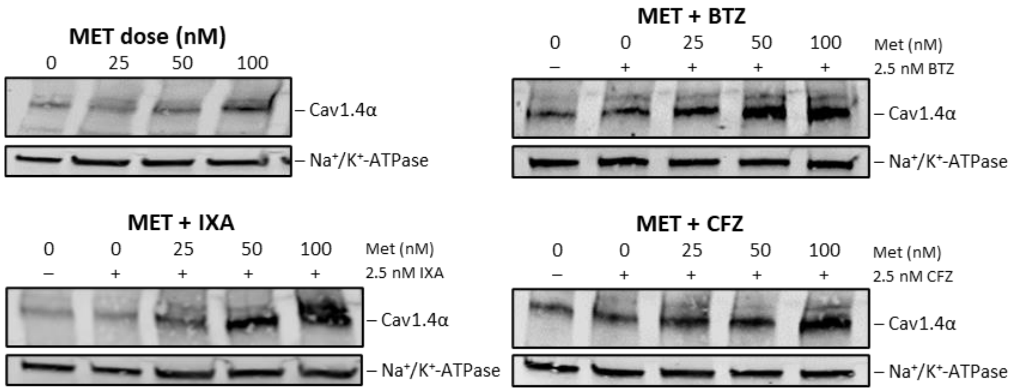

B

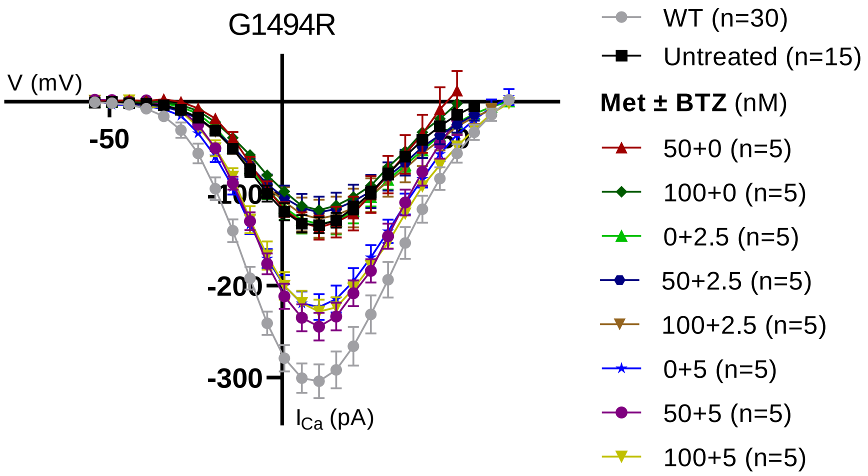

Fig. S5. Effect of Metformin (Met) and proteasome inhibitors (PIs) on $Ca_v1.4$ p.G1494R protein expression and channel function.

(A) Representative western blot of $Ca_v1.4$ p.G1494R expression following a 6h treatment with either metformin alone or in combination with three different PIs. The concentrations and combinations are indicated. **(B)** I/V plots of I_{Ca} for $Ca_v1.4$ p.G1494R treated with different combinations of Met and PIs for 6h. No combination significantly increased the peak I_{Ca}. Currents were evoked by 5 ms depolarisation from -80 to 80 mV with P/4 leak subtraction. The wild type $Ca_v1.4$ traces are an accumulation from multiple experiments (n=30). Each cell trace was recorded three times and an average was taken. Error bars represent mean ± S.E.M. Met, Metformin; BTZ, Bortezomib; CFZ, Carfilzomib; IXA, Ixazomib; WT, wild type.

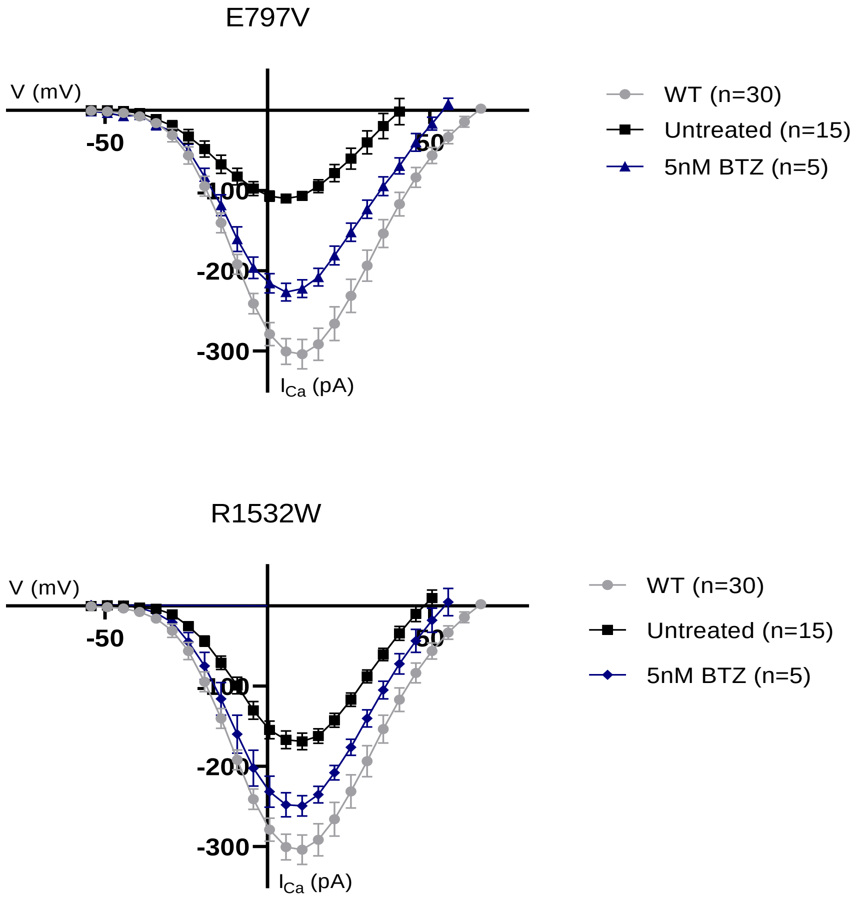

Fig. S6. Effect of Bortezomib (BTZ) on the function of Ca_v1.4 p.E797V and p.R1532W.

(B) I/V plots of I_{Ca} for Ca_v1.4 p.E797V and p.R1532W treated with Bortezomib (BTZ). BTZ increased the I_{Ca} currents for both mutations. Currents were evoked by 5 ms depolarisation from -80 to 80 mV with P/4 leak subtraction. The wild type Ca_v1.4 traces are an accumulation from multiple experiments (n=30). Each cell trace was recorded three times and an average was taken. Error bars represent mean ± S.E.M. BTZ, Bortezomib; WT, wild type.

Table S1. Plasmids used in this study.

Construct	Source
**pcDNA3.1_α_{1f} (wildtype)**	Prof Amy Lee (University of Texas)
**pcDNA3.1_$\alpha_2\delta$**	Prof Amy Lee (University of Texas)
**pUC18_β_3**	Prof Alexandra Koschak (University of Innsbruck)
**pcDNA3.1_α_{1f}_D119Y**	This study
**pcDNA3.1_α_{1f}_R290C**	This study
**pcDNA3.1_α_{1f}_G674S**	This study
**pcDNA3.1_α_{1f}_E797V**	This study
**pcDNA3.1_α_{1f}_D1097N**	This study
**pcDNA3.1_α_{1f}_N1434S**	This study
**pcDNA3.1_α_{1f}_P1491L**	This study
**pcDNA3.1_α_{1f}_G1494R**	This study
**pcDNA3.1_α_{1f}_K1506N**	This study
**pcDNA3.1_α_{1f}_R1532W**	This study

Table S2. Inhibitors used in this study.

Inhibitor	Pathway	Concentration	Supplier	Identifier
**Cycloheximide (CHX)**	Translation	20 µg/ml	Sigma-Aldrich	C1988
**MG132**	Proteasome	20 µM	Sigma-Aldrich	M7449
**Bortezomib (BTZ)**	Proteasome	2.5 - 25 nM	Adooq Bioscience	A10160
**Carfilzomib (CFZ)**	Proteasome	2.5 - 100 nM	Adooq Bioscience	A11278
**Ixazomib (IXA)**	Proteasome	2.5 - 100 nM	Adooq Bioscience	A10600
**Chloroquine phosphate**	Lysosome	50 µM	Sigma-Aldrich	PHR1258
**Ammonium chloride**	Lysosome	50 mM	Sigma-Aldrich	A9434
**Metformin (Met)**	–	25 – 100 nM	Adooq Bioscience	B1970-APE

Table S3. Primary antibodies used in this study.

Target	Host species	Dilution	Supplier	Identifier
**Ca_v1.4α (VDCCα1F)**	Mouse	1: 1000	Abnova	H00000778-A01
**Sodium-potassium adenosine triphosphatase (Na^+/ K^+-ATPase)**	Rabbit	1: 20,000	Abcam	ab76020

Table S4. Secondary antibodies used in this study.

Fluorophore	Target	Host species	Dilution	Supplier	Identifier
**IRDye-800CW**	Mouse IgG	Donkey	1: 5000	LI-COR	926-32213
**IRDye-680RW**	Rabbit IgG	Donkey	1: 5000	LI-COR	925-68072

Table S5. Summary of $Ca_v1.4$ VUS functional data.

The functional result collated for each $Ca_v1.4$ variant and the proposed class of mutation. Percentage changes are normalised and relative to the wild type protein and all *p* values are listed.

**Mutations**	**% Peak currents relative to wild type (*p* value)**	**% Protein expression relative to wild type (*p* value)**	**% Protein remaining after 8h with CHX relative to wild type (*p* value)**	**Mutation class**
Asp119Tyr	36.7 (p<0.0001)	64.5 (p<0.0.0008)	56.8 (p<0.0003)	Hypomorph
Arg290Cys	66.2 (p<0.0004)	78.1 (p<0.0319)	69.9 (p<0.0006)	Hypomorph
Gly674Ser	22.4 (p<0.0001)	88.3 (p<0.0479)	76.9 (p<0.0024)	Hypomorph
Glu797Val	39.8 (p<0.0001)	69.3 (p<0.0030)	35.5 (p<0.0001)	Hypomorph
Asp1097Asn	0 (p<0.0001)	78.4 (p<0.0010)	54.6 (p<0.0002)	Complete hypomorph
Asn1434Ser	0 (p<0.0001)	72.8 (p<0.0001)	46.3 (p<0.0002)	Complete hypomorph
Pro1491Leu	74.2 (p<0.0071)	63.7 (p<0.0004)	71.7 (p<0.0017)	Hypomorph
Gly1494Arg	40.3 (p<0.0001)	62.3 (p<0.0001)	39.4 (p<0.0001)	Hypomorph
Lys1506Asn	96 (p<0.4841)	98 (p<0.1161)	98 (p<0.1583)	Splice variant
Arg1532Trp	56.9 (p<0.0001)	49 (p<0.0001)	17.4 (p<0.0001)	Hypomorph
